# Study of Methods for Assessing Research Topic Elicitation and pRioritization (SMARTER): Study Protocol to Compare Qualitative Research Methods and Advance Patient Engagement in Research

**DOI:** 10.2196/resprot.7565

**Published:** 2017-09-07

**Authors:** Danielle C Lavallee, Bryan Comstock, Mary R Scott, Andrew L Avins, David R Nerenz, Todd C Edwards, Donald L Patrick, Sarah O Lawrence, Zoya Bauer, Anjali R Truitt, Jeffrey G Jarvik

**Affiliations:** ^1^ Surgical Outcomes Research Center Department of Surgery University of Washington Seattle, WA United States; ^2^ Department of Biostatistics University of Washington Seattle, WA United States; ^3^ Kaiser Permanente Northern California Oakland, CA United States; ^4^ Henry Ford Health System Detroit, MI United States; ^5^ Department of Health Services University of Washington Seattle, WA United States; ^6^ Comparative Effectiveness, Cost and Outcomes Research Center Department of Radiology University of Washington Seattle, WA United States

**Keywords:** patient involvement, qualitative research, research priorities, back pain

## Abstract

**Background:**

Involving patients as partners in research is a defining characteristic of patient-centered outcomes research (PCOR). While patients’ experiential knowledge of a health condition or treatment may yield research priorities not reflected by researchers and policy makers, the methods for identifying and effectively collaborating with patients are still evolving. Patient registries and crowdsourcing may offer ease of access and convenience to both researchers and patients. Surveys and focus groups, including online modalities, have been described for prioritizing research topics. However, little is known about how these different methods compare in producing consistent priorities and similar perceptions of engagement quality among participants.

**Objective:**

The aims of this study are (1) to compare how different engagement methods used to elicit patient priorities for research perform as measured by rankings for priorities generated and participant satisfaction; and (2) to determine characteristics of individuals choosing to participate in research prioritization activities.

**Methods:**

Participants in the Back pain Outcomes using Longitudinal Data (BOLD) patient registry, established to evaluate the natural history of back pain among individuals 65 years and older, and participants on the Amazon Mechanical Turk (MTurk) crowdsourcing platform, to provide input on priorities for research via a questionnaire, are invited. For BOLD participants, we subsequently randomize interested respondents to 1 of 3 interactive prioritization activities to further develop priorities: a Delphi panel, an online crowd voting activity, or an in-person facilitated prioritization activity using nominal group technique (NGT). Participants involved in each activity complete a survey to evaluate the quality of the experience and a subset of these participants discuss their experience further in an interview. Descriptive statistics are used to characterize the rankings produced by each method and compare the top 5 rated topics resulting from each prioritization activity. We use rank-ordered logistic regression models to identify associations of the ranked priority topics with baseline patient characteristics. We analyze responses to the evaluation using a mixed-methods approach wherein we tabulate responses to Likert-scale questions and use content analysis to enumerate themes emerging from interviews for the 3 activities.

**Results:**

In Phase I, we invite approximately 3000 BOLD participants and 500 Amazon MTurk workers to complete a research topic prioritization survey. Based on these results, we include additional topics into a subsequent prioritization survey. In Phase II, we invite BOLD participants to join 1 of 3 activities: 90 participants for Delphi panel, 100 participants for crowd voting, and 60 participants for focus groups. Of the Phase II participants, 30 will be interviewed to evaluate the activities.

**Conclusions:**

This study informs decisions about how to conduct outreach to patient registry participants for providing input on research priorities, how individuals 65 years and older wish to participate in engagement activities, and how different research prioritization methods compare in terms of rankings generated and participant satisfaction.

## Introduction

The direct involvement of patients as partners in research is a defining characteristic of patient-centered outcomes research (PCOR). Involvement of patients throughout the research process—starting with the identification of the research question—ensures that the research conducted centers on evidence gaps that patients face when making decisions about their healthcare. Without patient representation, research agendas do not align with information needs of greatest importance to patients [1]. Funding agencies, government organizations, and advocacy organizations at the local, national, and international levels often establish priorities for future research and funding using diverse approaches with varying levels of patient involvement in the process [2-6]. Despite the time and resources dedicated to this important effort, little is known about how different prioritization methods compare in participant experience and priorities generated.

One barrier cited for greater patient involvement is the ability to identify patients interested and able to participate in research activities [2]. Traditionally, representatives from formal patient advocacy organizations provide the patient perspective in priority-setting activities, yet such formal representation is not always available and may represent a different perspective than that of the broader patient community.

One example is low back pain (LBP), one of the most important causes of functional limitations and disability worldwide [7,8]. Despite the prevalence of LBP, national patient advocacy organizations focused on this health condition do not exist. In this scenario, as with other diseases and health conditions without formal patient organization representation, research agendas are often set without the patient perspective fully represented.

Patient registries provide an opportunity to address this gap. Patient registries are developed to collect data on a defined patient population with a specific disease or condition. To better understand the effectiveness, safety, and cost-effectiveness of interventions for older patients with low back pain, the Back pain Outcomes using Longitudinal Data (BOLD) study, funded in 2010 by the Agency for Healthcare Research and Quality, established a large, community-based registry of older patients with LBP [9]. Leveraging this research infrastructure provides one potential avenue for involving patients in topic prioritization, yet little is known about the feasibility or predictors of participation in this process. Newer approaches to involvement, such as social media and crowdsourcing platforms, are also emerging as ways to expand outreach and obtain input from broader audiences. For example, Amazon created a crowdsourcing Internet marketplace, Mechanical Turk (MTurk), which allows individuals to participate in activities—a number driven by research interests [10]. Further exploration of how these communities support PCOR is needed.

With Patient-Centered Outcomes Research Institute (PCORI) funding, we aim to compare different methods for obtaining input from patients on future research topics in both participant experience and priorities generated. We will test the hypotheses that the different methods produce similar rankings for research priorities but differ in participant-rated experience with methods with greater participant interaction receiving better ratings. This 2-phase study first assesses participant characteristics and research priorities from 2 populations: participants in the BOLD registry and participants from the MTurk platform. In the second phase, different interactive methods engaging patients in research prioritization among BOLD registry participants are compared.

## Methods

### Patient Engagement in the Research Process

Patient engagement is a core component of PCOR processes with the goal of improving the quality and relevance of research [4]. Patient engagement in this study occurs through direct involvement of a patient partner on the research team (Ms Scott), discussions with patient advisors at each site, and outreach to the CERTAIN Patient Advisory Network Back Pain Research Patient Advisory Group [11], a committee established by researchers at the University of Washington (UW) for the purpose of supporting patient engagement across a number of ongoing research initiatives, including BOLD. Our patient partner participates in all research team meetings as an equal member in all decisions. Patient partners at BOLD study sites assist with iterative study material development. Finally, input on study activities is obtained through quarterly meetings held with the CERTAIN Patient Advisory Network Back Pain Research Patient Advisory Group, a group of 10 individuals with back pain who convene to discuss a number of ongoing and developing research projects. Standing time on the agenda allows for study updates, requested input on study activities, and general discussion about results and findings.

### Study Overview

**Figure 1:**
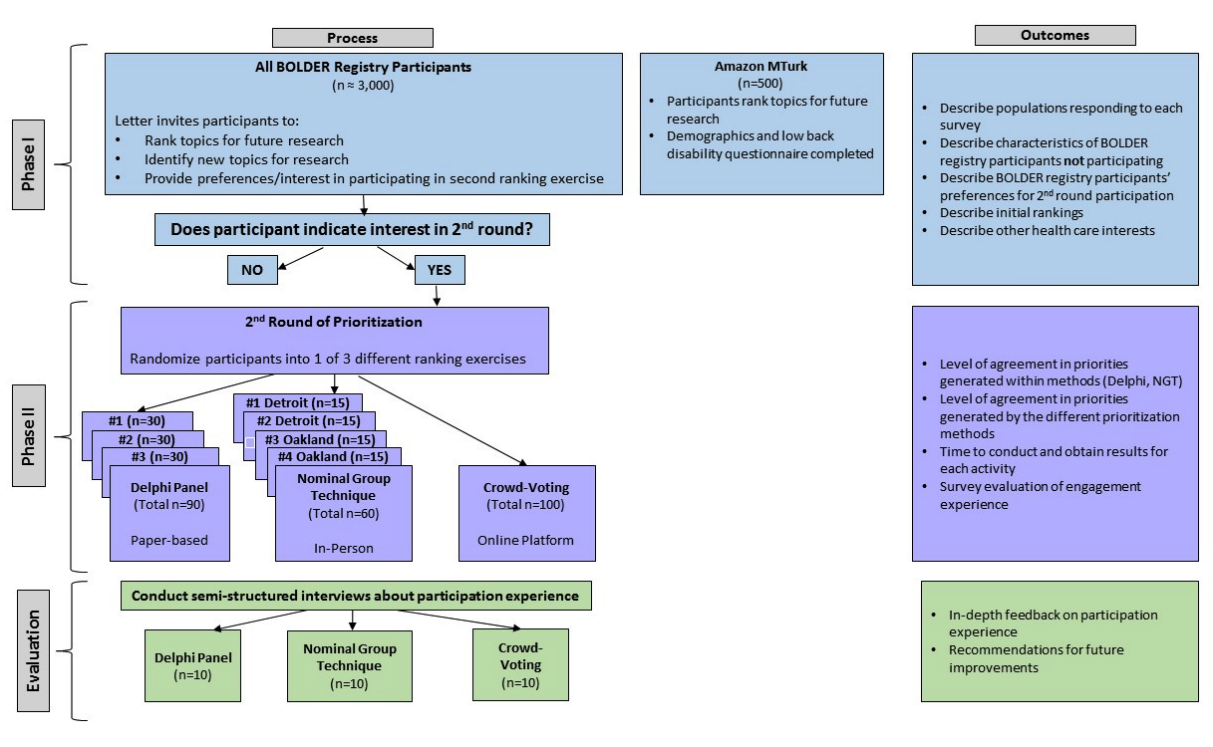
Study overview.

This is a 2-phase study (Figure 1). The first phase elicits research priorities from 2 different patient populations (the BOLD registry and MTurk) via questionnaire. The second phase uses random assignment of BOLD registry respondents from Phase I to participate in 1 of 3 subsequent prioritization activities: (1) focus group using nominal group technique (NGT), (2) modified Delphi process, and (3) online crowd voting. These methods vary in the level of interaction between participants and mode of involvement. During each activity, participants convene to review and prioritize the list of topics (both existing and newly generated) from Phase I.

### Institutional Review Board Approval

The institutional review boards (IRBs) at all collaborating institutions (UW, Henry Ford Health System [HFHS], and Kaiser Permanente, Northern California [KPNC]) reviewed and approved the protocols for this study.

### Phase I Study Procedures

#### Participant Eligibility and Recruitment

The BOLD registry consists of older adults with back pain. The inclusion and exclusion criteria for BOLD is presented in Textbox 1. Two of the original 3 BOLD clinical sites, KPNC and HFHS in Detroit, MI, participated in the Study of Methods for Assessing Research Topic Elicitation and pRioritization (SMARTER) study [9]. The sites represent diversity in patient demographics and clinical experience [9]. At the time of the SMARTER study initiation, a total of 4131 patients were enrolled in the BOLD registry (3164 at Kaiser and 967 at Henry Ford), with a 1-year follow-up retention rate of 85%. BOLD registry participants at the 2 participating sites (KPNC and HFHS) with current contact information completing 24-month follow-up are eligible to participate in the prioritization activity.

#### MTurk

The Amazon MTurk platform provides access to an online community interested in providing input on an array of activities, including research, requests for completion of basic tasks, and participation in opinion polls [10]. MTurk reports more than 500,000 registered users (MTurk workers) throughout the world.

MTurk workers with an active account registered in the United States are eligible to participate in the crowdsourcing prioritization activity. MTurk workers screen for the prioritization activity or human intelligence task (HIT) through a questionnaire accessed through the MTurk platform. The posted HIT to prioritize research topics in LBP will offer workers US $0.10 for completing the initial screening questionnaire and once approved, an additional US $0.75 to complete the prioritization task. Incentives are directly credited to the participants' Amazon account. Workers selecting to complete our HIT first complete the Roland Morris Disability Questionnaire (RDQ) [12], a validated measure for back pain, and the primary patient-reported outcome measure in the BOLD registry. Participants who score a 7 or greater on the RDQ are invited to continue and complete a topic prioritization activity.

**BOLD registry inclusion and exclusion criteria.**

Criteria

- Inclusion criteria
  - Age 65 years and greater
  - Primary care visit for back pain based on ICD9 code
- Exclusion criteria
  - Healthcare encounter for back pain within 6 months
  - Previously contacted for registry participation
  - Prior lumbar spine surgery
  - Developmental spine deformities
  - Inflammatory spondyloarthropathy
  - History of cancer within the past 5 years excluding non-melanomatous skin cancer
  - History of HIV within past 5 years
  - No telephone
  - Planning on leaving health system within the next 12 months
  - Unable to understand English
  - Severe mental impairment that would interfere with answering questions

#### Questionnaire Development

Materials developed for this study include the invitation for participation, instructions, and a prioritization questionnaire. Invitation letters include a short introduction to PCOR, explanation of the study goals, and a list of research topics for prioritization adapted from topics identified and published in 2013 by back pain primary care clinicians and researchers [13]. Instructions guide participants to review the list, provide (if desired) up to 5 additional topics not listed, and from this list, rank their top 5 research topic priorities. Participants are instructed to rank their highest priority with the number 1.

#### Data Collection

##### BOLD Registry

Research coordinators at each site approach recruitment by mailing questionnaires to all eligible participants enrolled from their respective health system. Participants have the option to submit responses via postage paid mail or to complete the questionnaire by phone. Participants not responding within 14 days of initial outreach (returned questionnaire or opt-out form) receive a follow-up phone call from BOLD research coordinators. All responses are entered into a secure and encrypted system maintained at the registry site.

##### MTurk

Eligible MTurk workers complete a topic prioritization questionnaire through a unique survey link. Participants review the list of priorities (identical to those provided to the participants from the BOLD registry), provide (if desired) up to 5 additional topics not listed and from this list, rank their top 5 research topic priorities. In addition to the topic prioritization, workers provide basic demographic information (Textbox 2). Inclusion of the unique MTurk identification code is optional but required for payment to the appropriate MTurk account. The HIT closes after 1 month or once 500 HITs are completed.

#### Data Management

The UW serves as the Data Coordination Center for the BOLD registry and serves in the same capacity for this study. The UW Data Coordination Center coordinates the recruitment and follow-up of study participants across sites and provides a common infrastructure for the management of study data. Research coordinators receive all returned questionnaires and enter data into Research Electronic Data Capture (REDCap) [14], a software platform specifically designed for electronic data capture in research studies.

### Phase II Study Procedures

#### Participant Eligibility and Recruitment

Participants from BOLD Phase I indicating willingness to participate in a second, interactive topic prioritization activity are eligible for randomization into 1 of 3 different activities: focus groups using NGT, a 2-round modified Delphi process, or an online crowd voting activity. We randomize eligible participants taking into account individual preference for activity. The MTurk platform prohibits the collection of identifiable information, thus, MTurk participants are excluded from Phase II activities [15].

**Demographic information collected for participants.**

Demographic information

- Age
- Sex
- Race
  - Caucasian
  - Black or African American
  - Asian
  - Native American Indian, Native Alaskan, Native Hawaiian or other Pacific Islander
  - Other
- Ethnicity
  - Hispanic
  - Non-Hispanic
- Education
  - Less than high school graduate
  - High school grad or obtained General Education Development (GED)
  - Some college
  - Vocational, technical, trade, or associate’s degree
  - 4-year college graduate
  - Professional or graduate degree
- Employment
  - Employed, full time
  - Employed, part time
  - Not employed, looking for work
  - Unable to work, not employed, not looking for work
  - Retired
- Marital status
  - Married
  - Living with a partner
  - Divorced/separated

UW research staff recruit participants randomized to each activity by phone approximately 6 weeks prior to the planned start of each activity. For recruitment calls, study staff describe the activity that the participant is invited to join, including the purpose, the goals of the activity, what occurs during the activity, the expected time commitment, the participants’ role in the activity, and incentives for participation. Recruitment continues until capacity is reached for all planned engagement activities. We exclude participants for Web-based engagement (ie, crowd voting) if they do not have ready access to a computer or an active email address. Research staff will contact consented participants at 2 weeks and again 2 days prior to the activity as a reminder of the event and to answer any questions.

#### Priority-Setting Activities Overview

Standard materials developed for all activities include a common-language overview of the project, goals for the activity, and instructions on the ranking process. In addition, each participant receives a full list of topics ranked during Phase I appended with new topics identified by participants. Excluding descriptions of and instructions for each specific activity, all preparation materials are designed for consistent messaging across all methods to reduce variation in external factors that could influence the experience or outcomes. All participants complete a consent form and brief demographic form prior to the prioritization activity (Textbox 2).

##### Focus Group with Nominal Group Technique

Focus group with NGT is a structured group discussion method used to generate consensus [16,17]. The method combines individual work and thought for idea generation with structured interactive group discussion [2,18]. In this manner, each individual develops and contributes ideas ensuring that no one perspective dominates the discussion or activity. We plan to hold in-person focus groups at each study site with up to 30 participants from each site (10 people per group). Priority lists will be produced in multiple rounds during the focus groups. We plan to audio-record and transcribe the focus groups. Participants receive US $100 at the conclusion of the activity for an expected 4 hours total time for participation, including preparation time.

##### Modified Delphi

Modified Delphi uses a series of questionnaires with controlled feedback to systematically and efficiently obtain input from respondents with desired knowledge and experience in a given area [16,17,19]. Unlike focus groups with NGT, the Delphi method does not require in-person interaction, rather it uses written responses to exchange ideas and information [18]. For the purpose of this activity, we adopt a common practice and modify the method to 2 rounds in which the initial questionnaire provides topics for ranking rather than generating them de novo. We plan to conduct 3 modified Delphi activities among 3 different groups, with each group consisting of 30 participants. Conducting 3 separate modified Delphi activities allows for assessment of how this method performs across randomized samples. In the first round, we provide participants with a list of prioritized topics plus newly identified topics from Phase I with instructions to indicate importance of individual topics using a 5-point Likert scale. When all completed questionnaires are received back from all participants within a group, UW investigators summarize the findings and develop a second questionnaire to send back to participants. During the second round, participants review the prioritized list generated by the group in the first round and revise their rating of the topics, should they choose to do so. Participants receive US $50 at the conclusion of the activity for an expected 2 hours total time for participation, including preparation time.

##### Online Crowd Voting

Online crowd voting uses an Internet community platform allowing participants the opportunity to submit ideas, vote on existing ideas, and interact with others through online discussion. The crowd voting activity brings together up to 100 people with LBP for online discussion and voting through a secure Internet-based program called IdeaScale [20]. This activity occurs over the course of 1 month. Participants create accounts to access the online private community allowing the opportunity to vote on topics for LBP research, discuss topics that are posted, and share new topics. Participants will be asked to sign in to the community at least twice over the course of the 1-month time period. Minimal group moderation led by study staff will occur over the course of the activity to support community involvement. Participants receive US $25 at the conclusion of the activity for an expected 1-hour total time for participation including preparation time.

#### Evaluation

Participants evaluate the quality of experience and perceived effectiveness of each method for generating topics. Evaluation questions assess how effective each method performs in meeting overarching goals of PCOR as trustworthy, fair, balanced, legitimate, respectful, and accountable [3,21]. Questions evaluating process and outcomes include items adapted from work by Van De Ven and Delbecq [18] as part of a comparative effectiveness analysis of group decision-making processes, participant ratings on perceived satisfaction with the number of topics generated, and perception that the group process is an effective way to provide input. We plan to include open-ended questions to elicit input on the aspects that participants liked most and least for each engagement activity and if participation resulted in a change in priorities ranked. Participants of the modified Delphi process and crowd voting activity receive the questionnaire via mail or the Internet at the end of the prioritization activity. Individuals participating in the focus group receive the evaluation questionnaire in person immediately following the activity.

Finally, participants are invited to provide additional feedback via a phone interview to elicit in-depth feedback on experience and satisfaction with involvement. Up to 30 interviews with participants from each of the different interactive engagement activities (ie, 10 Delphi participants, 10 focus group participants, and 10 crowd voting participants) participate in interviews. We select interviewees representing a range (negative and positive) of responses from the evaluation questionnaire. We record interviews, with consent, for transcription purposes.

#### Data Management

Data collected in Phase II includes focus group transcripts, Delphi surveys, online crowd voting activity, evaluation surveys, demographic surveys, and evaluation interview transcripts. Audio recordings and transcriptions of focus group discussions and evaluation interviews are stored on a secure and encrypted system maintained at UW. UW research staff enter Delphi survey data directly into a REDCap database. IdeaScale exports data from the online community at the conclusion of the activity for upload into the study database.

### Analysis

#### Phase I

We plan to describe the characteristics of the populations participating in each prioritization activity, including the initial paper-based questionnaire mailed to all BOLD registry participants and MTurk activity. We will evaluate, compare, and describe the characteristics of BOLD registry patients who elect to participate further compared to those who do not. Patient characteristics will include geographic location, age group (65 to 74, 75 to 80, greater than 80 years), gender, pain as measured by the 0 to 10 point numeric rating scale (NRS), disability as measured by the RDQ, education, marital status, and duration of pain.

#### Phase II

We will describe the characteristics of the populations participating in each prioritization activity (focus group with NGT, modified Delphi process, and crowd voting). To identify potential bias among those who choose to participate versus those who do not, we will analyze the characteristics of non-responders in the different groups compared to responders across Phase II activities. We will evaluate the primary hypothesis that the different methods produce similar priorities by comparing the top 5 highest rated topics resulting from each prioritization activity using descriptive statistics. We will use rank-ordered logistic regression models to identify associations of the ranked priority topics with baseline patient characteristics. In addition, we will report on the number and type of new topics identified by respondents in each activity.

We will evaluate the secondary hypothesis that participants will prefer methods with greater participant interaction as defined both by in-person interaction and ability to engage in discussion with other participants (focus groups with NGT greater than online crowd voting greater than modified Delphi process greater than mailed survey) through analysis of Likert-scale questions using descriptive statistics for each activity. We will conduct a directed content analysis of transcribed interviews to better understand participant experiences and perceptions of prioritization activities.

## Results

**Figure 2:**
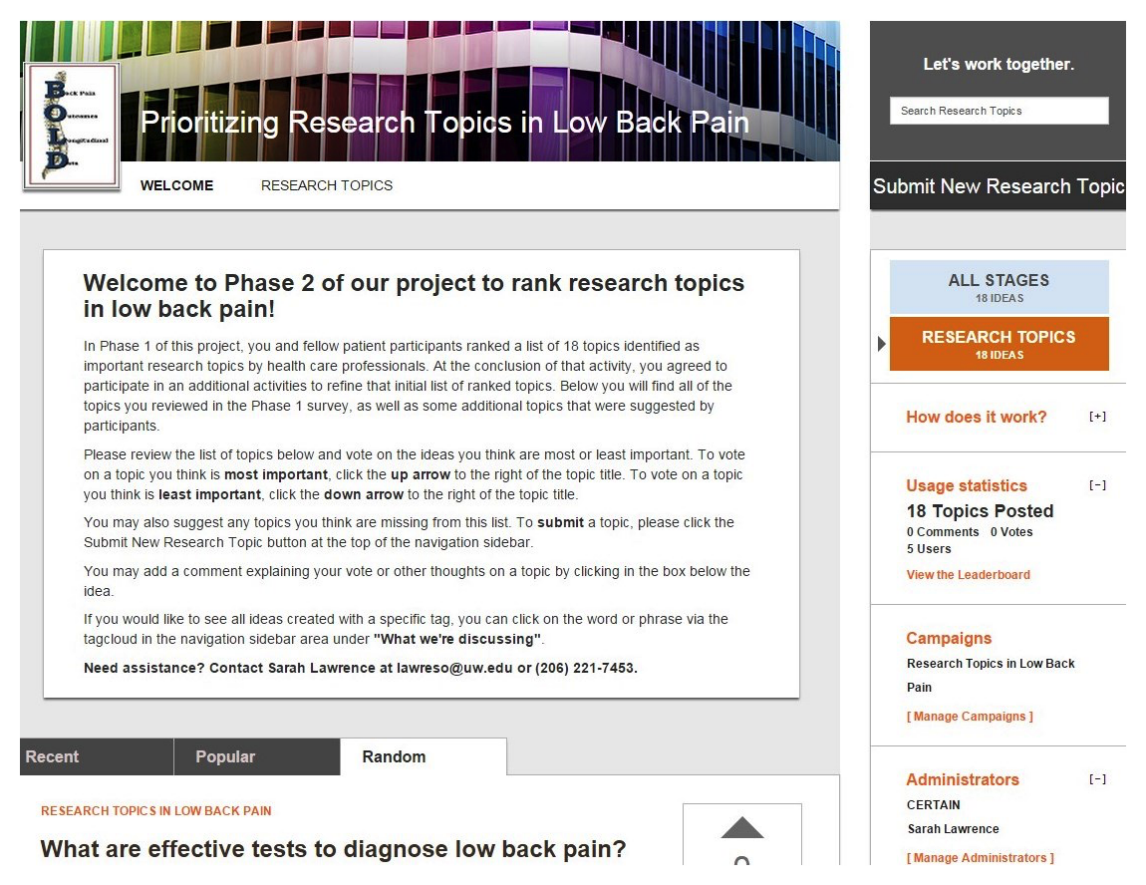
Screenshot of the crowd voting platform.

We provide an overview of our anticipated recruitment (Figure 1). In Phase I, we will invite approximately 3000 BOLD participants and 500 Amazon MTurk workers to complete a research topic prioritization survey. Based on these results, we will include additional topics into a subsequent prioritization survey. In Phase II, we will invite BOLD participants to join 1 of 3 activities: 90 participants for Delphi panel, 60 participants for focus groups, 100 participants for crowd voting (Figure 2). Of the Phase II participants, 30 will be interviewed to evaluate the activities.

## Discussion

### Principal Findings

This study assesses how different methods perform in generating reproducible research prioritization lists, and perhaps more importantly, in participants’ perception of the quality of the engagement experience. Such comparative studies are rare [18]. Two methods—the focus groups with NGT and modified Delphi process—are well described in the literature and are among the most commonly used methods [2,16,17]. The third, crowd voting, is a novel approach and reflects increasing interest in how Internet-based platforms may enhance survey methods to reach a large and diverse population to generate data while allowing for group interaction [2,22]. This study allows for comparison across different prioritization methods in generating similar results and perceptions of participant’s experience.

The intent of our proposed study is to add to the evidence to further support the conduct of priority-setting activities involving patients to inform the development and planning of research agendas. This is an area of growing focus. For example, the James Lind Alliance, a United Kingdom-based initiative established in 2004, brings together patients, caregivers, and clinicians for the purpose of identifying and prioritizing research priorities [23]. The PCORI brings together patients, caregivers, clinicians, and other healthcare stakeholders to identify priorities to shape areas for research funding. Our hope is to inform new methodological standards on involving patients in research activities and for topic prioritization, important building blocks for PCOR.

Two methods proposed in this study—crowdsourcing and crowd voting—are innovative. Crowdsourcing via the Internet is appealing in its ability to rapidly obtain responses from a broad and potentially diverse population [22,24]. Crowd voting, one example of crowdsourcing using open-source platforms, allows for interaction among participants through polling and open comments. This approach invokes transparency, as participants are able to view the activity and results as they unfold. Crowdsourcing requires minimal infrastructure to develop and permits rapid deployment [24]. In a previous study soliciting participants from MTurk, 500 eligible responses were obtained within 1 week [24]. These features make it appealing as a substitute for traditional survey-based methods that often require significant resources and time for distributing and collecting responses. Developing evidence using this novel approach and comparing it to older methods will advance and improve PCOR methods by incorporating emerging technologies into the research armamentarium. Further, if the PCOR field is going to embrace new technologies, we need to understand how they operate, particularly in older populations.

This study also explores how patient registries facilitate patient input and involvement in research outside of consented activities. Patient registries are organized to systematically collect data on a defined population to support clinical, research, and/or policy endeavors. When properly designed, registries provide important insight on the natural progression of disease and allow for assessment of the effectiveness or post-market safety of medications or devices as examples. Registry organizers and participants dedicate valuable time and effort to develop these important resources—learning with and from participants to support future work seems a natural next step.

Patient engagement is a core component of PCOR with the goal of improving the quality and relevance of research. Early involvement of our patient-partner during the research proposal development shaped early versions of the study protocol. Subsequent to receiving funding, her participation in research team meetings to further refine the protocol and design directly shaped the approach for recruitment and retention. For example, early plans included a token incentive of US $1 along with the initial prioritization questionnaire to participants. Based on Ms Scott’s input that this could demonstrate a perceived value to the importance of a patient’s perspective, the decision was made to instead place the funds towards staff-led outreach. Further, her involvement shaped the patient-facing materials. An important change made based on Ms Scott’s observations included a reorder of the listed priorities presented in the questionnaire. Initial drafts of the topics started with content about employment and work-related issues. As a retired person herself, Ms Scott recommended we change the order of priorities to avoid initial participant impressions that the questionnaire is not relevant as the majority of BOLD participants are over 70 years of age and no longer working. Other recommended changes to initial drafts included reducing the number of pages, increasing font size, clarifying instructions, and reducing technical terminology to improve comprehension.

One limitation of patient registries is the defined inclusion and exclusion criteria. The BOLD registry includes only individuals 65 years and older from integrated healthcare systems and thus, findings from the prioritization activities may not reflect experiences of younger individuals receiving care in different environments and health systems. Conducting a prioritization activity among non-BOLD registry participants (MTurk) allows for assessing generalizability and comparability with other methods. Mailed surveys and MTurk are similar in that participants are invited to participate to rank research priorities void of interaction with other participants. In this study, one difference is that for BOLD registry participants, the prioritization activity is conducted using traditional paper-based survey methods whereas MTurk is conducted online and is open to a broader and more heterogeneous audience for participation. Differences may exist between individuals choosing to participate versus those who do not. While we have the ability to assess these differences among BOLD registry participants, similar demographic and clinical information is not available for the MTurk population. Further, the MTurk platform policies prohibit collecting personal information, thus involving MTurk participants in Phase II activities is not possible. This limits the use of crowdsourcing platforms, such as MTurk, to non-interactive activities.

### Conclusion

This study provides a unique and rare opportunity to compare qualitative research methods, and to our knowledge, is the first study assessing such methods for the purposes of advancing patient engagement in research. Findings from the SMARTER study will provide funders, researchers, policy-makers, and organizations involving patients in the process of generating and prioritizing research evidence about how different approaches compare.

## Abbreviations

BOLD: Back pain Outcomes using Longitudinal Data
HFHS: Henry Ford Health System
HIT: human intelligence task
KPNC: Kaiser Permanente, Northern California
LBP: low back pain
MTurk: Mechanical Turk
NGT: nominal group technique
PCOR: patient-centered outcomes research
PCORI: Patient-Centered Outcomes Research Institute
RDQ: Roland Morris Disability Questionnaire
REDCap: Research Electronic Data Capture
SMARTER: Study of Methods for Assessing Research Topic Elicitation and pRioritization
UW: University of Washington
